# Expression of the proapoptotic protein Bid is an adverse prognostic factor for radiotherapy outcome in carcinoma of the cervix

**DOI:** 10.1038/sj.bjc.6602344

**Published:** 2005-02-01

**Authors:** M M L Green, G J Hutchison, H R Valentine, R J Fitzmaurice, S E Davidson, R D Hunter, C Dive, C M L West, I J Stratford

**Affiliations:** 1 Experimental Oncology Group, School of Pharmacy and Pharmaceutical Sciences, Coupland III, University of Manchester, Oxford Road, Manchester M13 9PL, UK; 2 Academic Department of Radiation Oncology, University of Manchester, Christie Hospital NHS Trust, Wilmslow Road, Manchester M20 4BX, UK; 3 Department of Histopathology, Clinical Sciences, Manchester Royal Infirmary, Oxford Road, Manchester M13 9WL, UK; 4 Department of Clinical Oncology, Christie Hospital NHS Trust, Wilmslow Road, Manchester M20 4BX, UK; 5 Cancer Research UK Cellular and Molecular Pharmacology Group, Paterson Institute of Cancer Research, Wilmslow Road, Manchester M20 4BX, UK

**Keywords:** Bcl-2 family, cervix carcinoma, prognosis, metastasis, radiotherapy

## Abstract

The Bcl-2 family of apoptotic regulators is thought to play an essential role in cancer development and influence the sensitivity of tumour cells to radiotherapy. Bid is an abundantly expressed Bcl-2 family protein playing a central role in various pathways of apoptosis by integrating and converging signals at the mitochondria. The relevance of apoptotic modulation by Bcl-2 and related proteins in tumour development and radiation response for human tumours remains undefined. Therefore, a study was made regarding the expression of Bid in patients with locally advanced cervix carcinoma who received radiotherapy. Bid expression was assessed using immunohistochemistry in pretreatment archival biopsies from 98 patients. The data were correlated with clinicopathologic characteristics and treatment outcome. Pretreatment tumour radiosensitivity data were available for 60 patients. Strong Bid expression was associated with a patient age less than the median of 52 years (*P*=0.034) and poor metastasis-free survival. In multivariate analysis, after allowing for stage, Bid expression was a significant prognostic factor for both disease-specific and metastasis-free survival (*P*=0.026). It is concluded that strong tumour Bid expression is associated with poor outcome following radiotherapy regardless of intrinsic tumour cell radiosensitivity, and is adverse prognostic for disease-specific and metastasis-free survival in younger patients.

Radiotherapy is the main treatment modality for locally advanced carcinoma of the cervix. In spite of the reduction in incidence and mortality in Europe and the USA, this disease remains one of the major causes of female cancer-related deaths worldwide. Defective apoptotic mechanisms may contribute to both malignant progression and radiation resistance of this tumour (Thompson, 1995; Haimovitz-Friedman *et al*, 1996). The Bcl-2 family of apoptotic regulators is believed to play an essential role in cancer development and also to influence the sensitivity of tumour cells to chemo- and radiotherapy. However, the relevance of apoptotic modulation by Bcl-2 and related proteins in tumour development and radiation response for human tumours remains undefined (Brown and Wouters, 1999; Belka and Budach, 2002; Coultas and Strasser, 2003).

Members of the Bcl-2 family include proapoptotic proteins such as Bid, Bak, Bax and Bad, and antiapoptotic proteins such as Bcl-2 and Bcl-X_L_. They are related by homology domains, known as BH domains 1–4, which are crucial for protein function and the intermolecular association of family members. Apoptotic signals, from intrinsic or extrinsic stimuli, are regulated via the relative actions and interactions of prosurvival and prodeath Bcl-2 proteins (Chao and Korsmeyer, 1998, Kelekar and Thompson, 1998; Korsmeyer, 1999). The signals lead to the release of apoptogenic factors (such as cytochrome *C* from mitochondria), proteolytic caspase cascade activation and ultimately cell death (Gross *et al*, 1999; Strasser *et al*, 2000; Cory and Adams, 2002).

Bid is an abundantly expressed BH-3 domain only protein that plays a central role in various pathways of apoptosis by integrating and converging signals at the mitochondria (Wang *et al*, 1996; Footz *et al*, 1998; Li *et al*, 1998; Luo *et al*, 1998; Strasser *et al*, 2000; Cory and Adams, 2002; Krajewska *et al*, 2002; Parone *et al*, 2002; van Loo *et al*, 2002). Bid is known to bind and antagonise Bax and Bak, at least one of which is absolutely required for the release of mitochondrial apoptogens, and induces apoptosis by coupling the proapoptotic activities of these proteins to the mitochondrial surface (Li *et al*, 1998; Luo *et al*, 1998; Desagher *et al*, 1999; Korsmeyer *et al*, 2000; Eskes *et al*, 2000; Wei *et al*, 2000, 2001; Cheng *et al*, 2001; Zong *et al*, 2001; Degli-Esposti, 2002). The best characterised signalling activity of Bid takes place following death receptor activation. Stimulation of death receptors such as Fas, TNFR and TRAIL by their respective ligands results in Bid activation in many cell systems (Li *et al*, 1998; Luo *et al*, 1998; Yamada *et al*, 1999; Degli-Esposti, 2002; Tafani *et al*, 2002). In response, caspase 8 is activated and subsequently cleaves Bid to form truncated Bid (tBid). Truncated Bid is considered to be the active form of the molecule that relocates to the mitochondria and promotes the release of apoptogenic factors (Li *et al*, 1998; Luo *et al*, 1998; Gross *et al*, 1999; Wei *et al*, 2000; Degli-Esposti, 2002), although full-length Bid can also induce apoptosis (Tafani *et al*, 2002; Sarig *et al*, 2003). Activation of Bid may also occur independently of death ligand stimulation, such as in the cytotoxic action of certain chemotherapeutic agents, or following activation of caspase 8 in response to intrinsic apoptotic stimuli (Sun *et al*, 1999; Tang *et al*, 2000; Sax *et al*, 2002). Bid is also directly cleaved by granzyme B, a protease delivered into target cells by cytotoxic T cells and natural killer cells as an immune-surveillance response (Barry *et al*, 2000; Heibein *et al*, 2000; Sutton *et al*, 2000). Thus, Bid has a central role among the Bcl-2 family members. It connects the intrinsic and extrinsic pathways for apoptosis, acts agonistically upon other pro-apoptotic Bcl-2 proteins, and plays an important role in apoptosis mediated by immune-surveillance mechanisms.

The aims of the present study were to investigate the expression of Bid in archived pretreatment biopsies of cervix squamous cell carcinoma, and to examine the results in relation to inherent tumour radiosensitivity as measured by SF2 (West *et al*, 1993, 1997), tumour pathological characteristics and clinical outcome following treatment with radiotherapy.

## MATERIALS AND METHODS

### Patients

Patients had histologically proven locally advanced squamous cell carcinoma of the cervix (FIGO stage Ib–IVa) and were registered at the Department of Oncology, Christie Hospital between 1987 and 1993. They were a consecutive series who received radiation therapy with curative intent and for whom a biopsy was taken for research. Patients were treated according to previous standard techniques of the Manchester School as described elsewhere (Pointon, 1991) before cisplatin was introduced as a concurrent treatment. The South Manchester Ethical Committee granted approval and prior informed consent was given by patients for tumour biopsies to be taken for research purposes at the time of their staging examination under anaesthesia. Clinical details of patient age, disease stage and tumour grade are listed in Table 1. Treatment outcome assessment was by review in specialist oncology clinics, and from questionnaires sent to general practitioners. The median followup time for surviving patients was 60 months (range 17–101 months). Sites of disease relapse were identified clinically and radiologically, and where appropriate confirmed on biopsy. Disease-specific survival is the time from start of radiotherapy to death due to cancer. Recurrence-free survival is the time from start of radiotherapy to cancer recurrence within the radiation field, and metastasis-free survival is the time to recurrence outside the radiation field.

For some of the patients included in the study, data were available from previous published work on tumour radiosensitivity (West *et al*, 1993, 1997). Radiosensitivity was measured on pretreatment tumour biopsies using an *in vitro* clonogenic assay to obtain surviving fraction at 2 Gy radiation (SF2).

### Immunohistochemical staining for Bid

**Figure 1:**
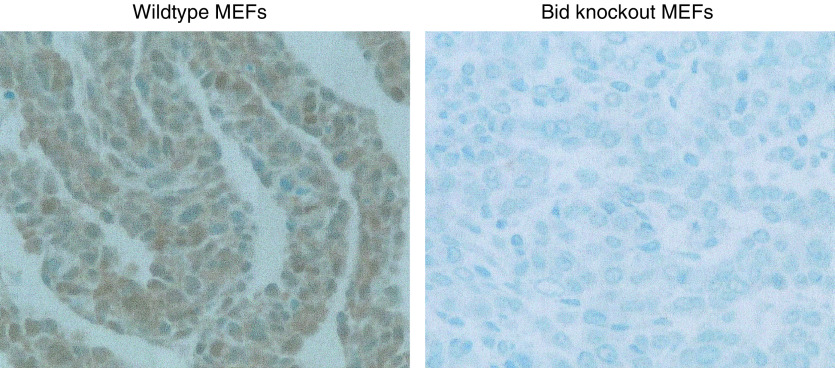
Photomicrographs of wildtype and Bid knockout mouse embryonic fibroblast (MEF) cell pellets demonstrating specific immunoreactivity.

After deparaffinisation of tumour sections, microwave antigen retrieval was performed in 10 mM citrate buffer (pH 6.0) and endogenous peroxidase was blocked with 3% methanolic hydrogen peroxide. A standard DAB-based immunostaining procedure employing an avidin–biotin complex reagent (DAKO) was used to detect Bid protein after application of specific goat anti-Bid antibody (R&D Systems, UK) at 1 *μ*g *μ*l^−1^ overnight at 4°C. Sections prepared from cell pellets of wild-type and Bid knock-out mouse embryonic fibroblasts (Yin *et al*, 1999) were used as controls for antibody specificity (Figure 1). The negative controls were goat IgG preimmune fraction (Alpha Diagnostic International Inc.) also at 1 *μ*g *μ*l^−1^, and PBS. Several control slides of predetermined Bid stain intensity were included in each batch to eliminate batch-to-batch variability. The presence of negative and positively stained nontumour cell types with consistent stain intensity within the cervical tissues served as further internal controls for stain specificity and fidelity.

### Assessment of Bid staining

**Figure 2:**
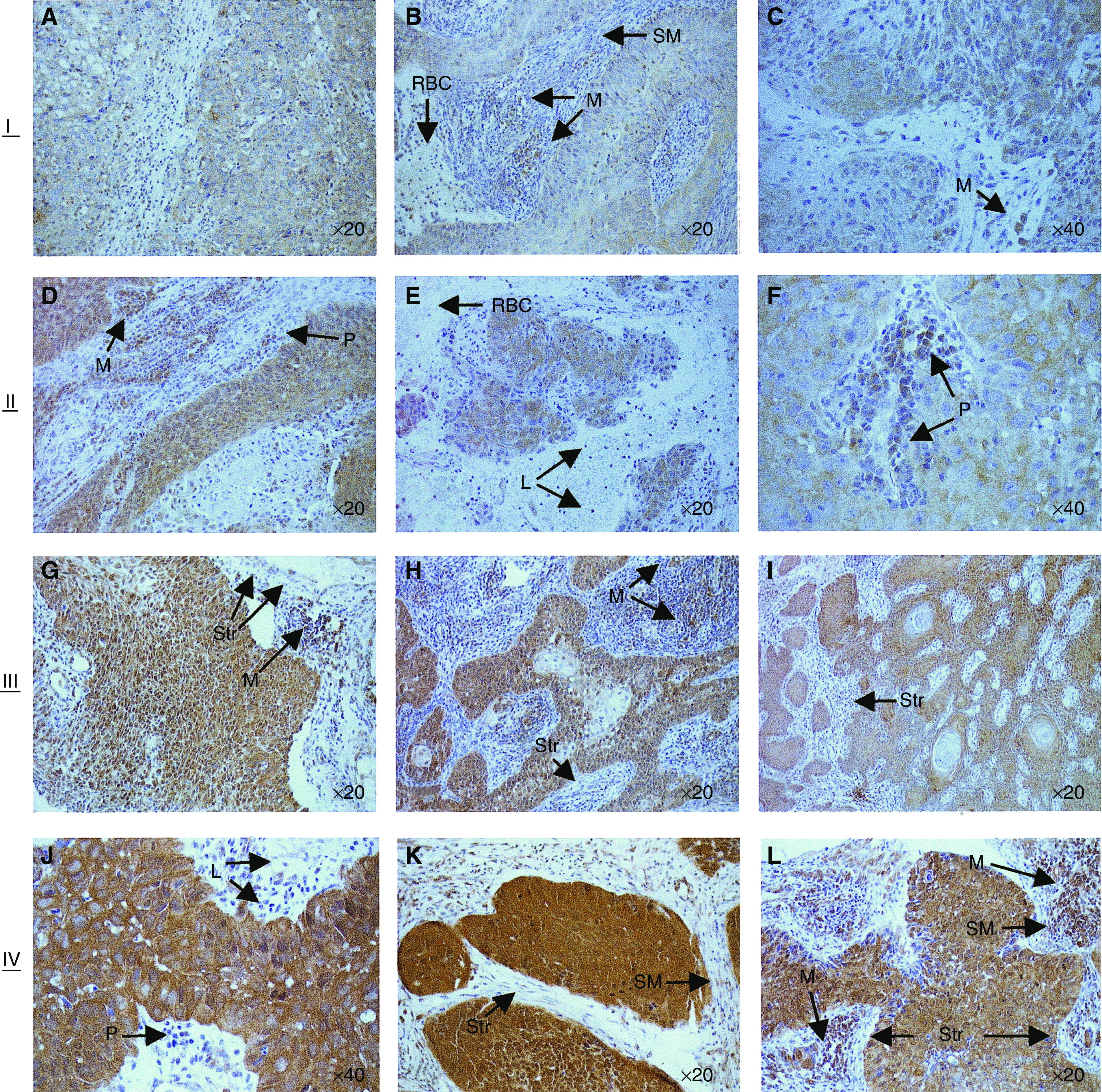
Photomicrographs of cervix carcinoma sections depicting Bid stain categories. I=lightest staining (**A**–**C**); II=weak staining (**D**–**F**); III=moderate staining (**G**–**I**); IV=strongest staining (**J**–**L**). Macrophages (M), neutrophils (N), plasma cells (P), smooth muscle cells (SM) were immunopositive: stromal cells (Str), red blood cells (RBC) and lymphocytes (L) were negative.

Tumour sections were assessed for the extent and intensity of Bid expression specifically within tumour cells without the observer's knowledge of clinical data. Necrotic areas, stroma and non-neoplastic epithelial cells were ignored. For the majority of tumours, 90–100% of the tumour cells were Bid positive. However, intensity of Bid stain varied from biopsy to biopsy and within tumour cells of individual biopsy sections. A comparative semiquantitative scoring index recommended for immunohistochemical biomarker validation was used to reflect the intensity of staining of individual cells and the percentage of cells that stained at different intensities (National Cancer Institute: Requirements for Pharmacokinetic and Biomarker Methods Development http://www3.cancer.gov/prevent ion/pio/biomarkers.pdf). Bid stain intensity was weighted as either absent (0), very weak (1), weak (2), moderate (3) or strong (4) and, for each biopsy section, the estimated percentage of tumour cells (to the nearest 10%) that expressed Bid at each intensity was assessed and multiplied by the corresponding weighted intensity value. These values were added to obtain a final score for each biopsy with a theoretical range of 0–400. The frequency distribution of the final scores was normal and quartiles were used to categorise scores (I–IV) for statistical purposes. Examples from each of these categories are illustrated in Figure 2.

### Statistical analysis

The reproducibility and concordance of scores were tested by Spearman's rank correlation and Altmann–Bland plot, respectively. The distribution of Bid score in relation to patient and tumour characteristics was investigated using either the Mann–Whitney *U* rank sum test or Kruskall–Wallis test to analyse the variance, and correlations were performed using Spearmans rank method. Survival analysis was performed by the Kaplan–Meier method, and prognostic factors were assessed by log-rank analysis. Univariate and bivariate analyses were made of disease-specific survival, local recurrence-free survival and metastasis-free survival. Bid expression, patient age, tumour stage, grade and SF2 were used to stratify patients. A stepwise multivariate Cox regression analysis was also performed to test the independence of putative prognostic parameters within the data set. All tests were two-sided and a significance level of *P*⩽0.05 was used throughout. All analyses were performed with SPSS version 10 for Windows (SPSS Inc., Chicago, IL, USA).

## RESULTS

### Immunohistochemical expression of Bid

Bid immunostaining demonstrated a granular, cytoplasmic pattern consistent with the cytoplasmic/mitochondrial/intercellular membrane association of Bid (Coultas and Strasser, 2003). Weak to moderate Bid immunoreactivity was detected throughout the normal residual stratified squamous epithelium of the biopsy specimens. Strong immunoreactivity was found in macrophages, moderately positive staining in neutrophils and plasma cells, and weak staining in smooth muscle cells of the cervical stroma and some vessel walls. Stromal cells, red blood cells and lymphocytes did not stain. Consistent intensity of Bid stain was obtained for the different normal cell types, whereas tumour cell Bid stain intensity varied (Figure 2). These observations are in agreement with a previous study of Bid expression in normal and malignant tissues that used alternative antibodies to detect Bid protein (Krajewska *et al*, 2002).

### Inter- and intraobserver reproducibility of scoring system

Tumour sections were scored twice by the first observer (MG) and ranged from 100 to 380. A significant correlation was obtained between the two scores (*r*=0.92, *P*<0.001) with an Altman–Bland plot equation of *y*=−0.0029*x*−2.058 showing good concordance. A significant correlation was obtained (*r*=0.93, *P*<0.001) when a series of 35 randomly selected tumour sections were scored by a second observer (GH).

### Distribution of patients according to Bid expression

Table 1 summarises the distribution of patients according to tumour Bid expression and clinical parameters. There was a significant difference in tumour Bid expression for patients stratified by the median age of 52 years, with a higher expression in younger women (*P*=0.034). This finding was supported by a weak but significant inverse correlation between patient age and Bid expression (*r*=0.27, *P*=0.007). There was no relationship between the level of Bid expression and disease stage, differentiation status and tumour radiosensitivity (SF2).

### Bid expression and radiotherapy outcome

**Figure 3:**
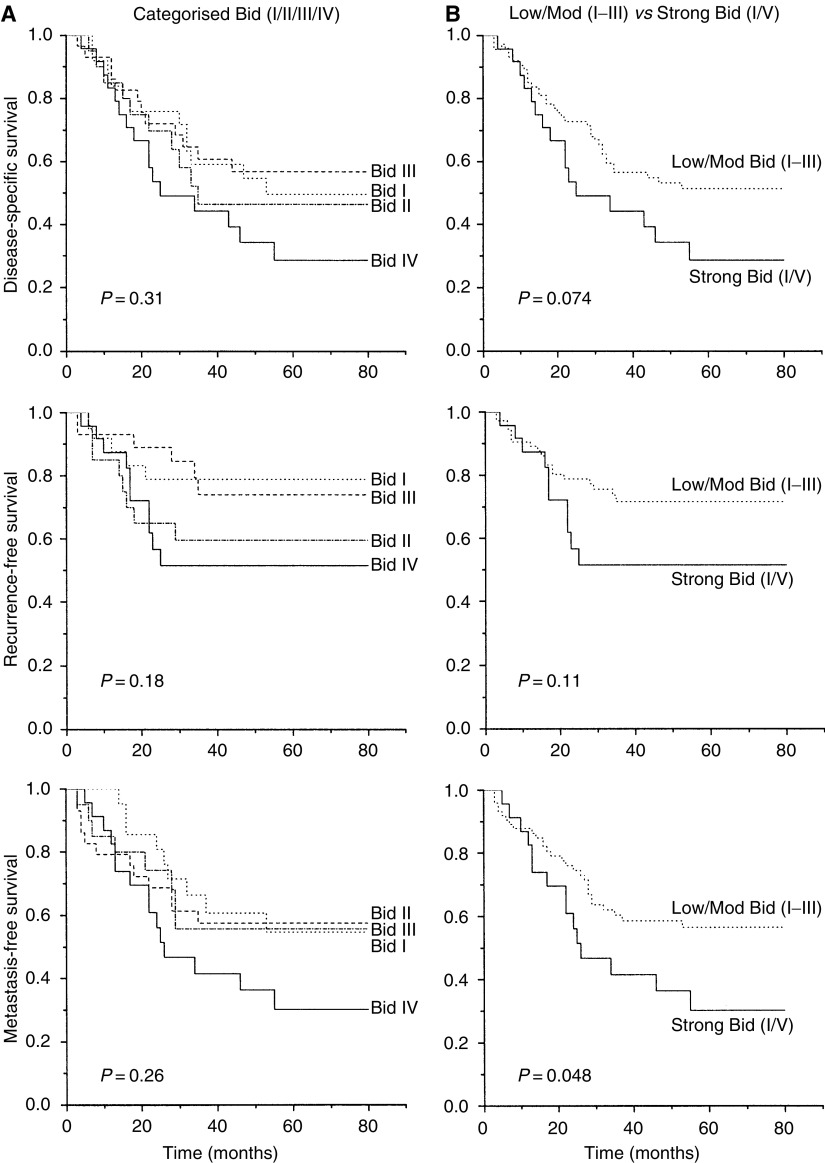
Bid expression and patient outcome. Disease-specific, recurrence-free and metastasis-free survival in relation to Bid expression in 98 patients with squamous cell carcinoma who underwent radiation therapy. Bid expression was categorised I–IV (**A**) and subsequently analysed with categories I–III grouped separately from category IV (**B**).

**Figure 4:**
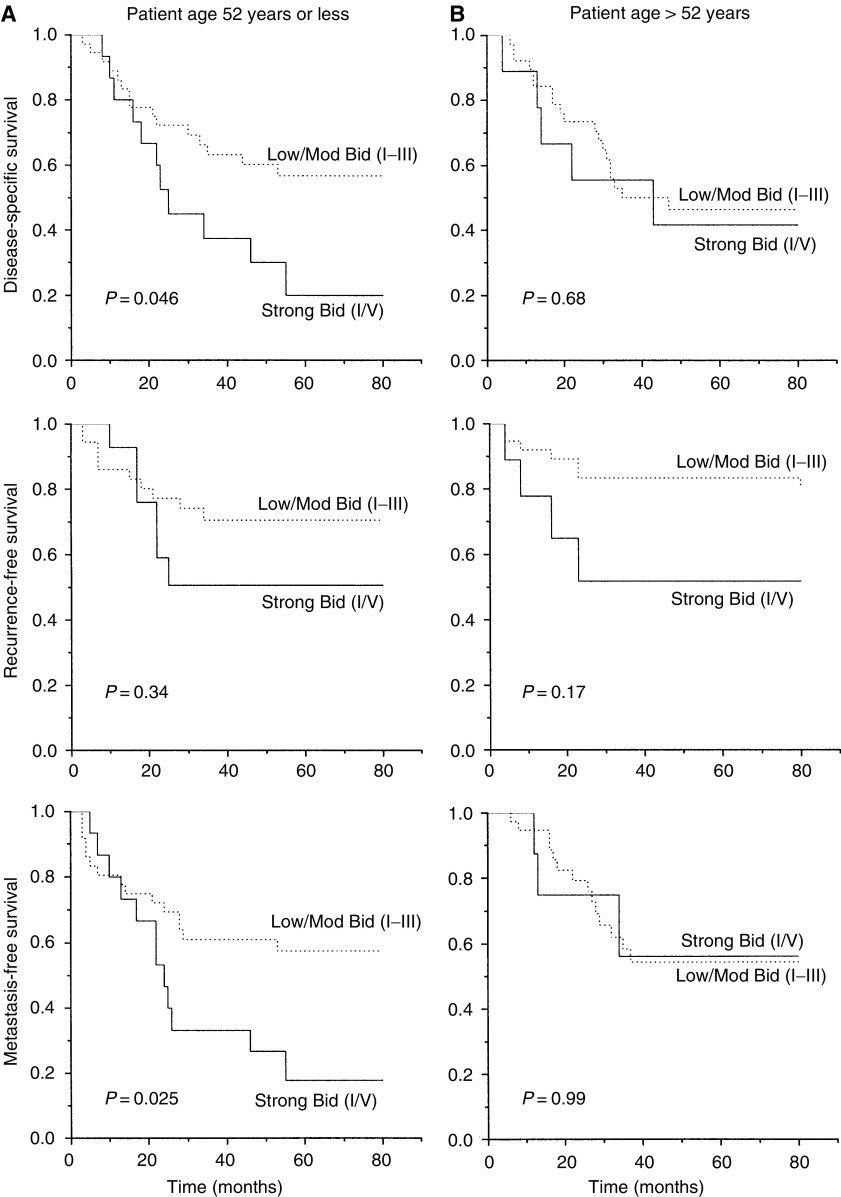
Patient age and outcome in relation to Bid expression. Patients were stratified according to median age (⩽52 years and >52 years) and grouped by Bid expression.

**Figure 5:**
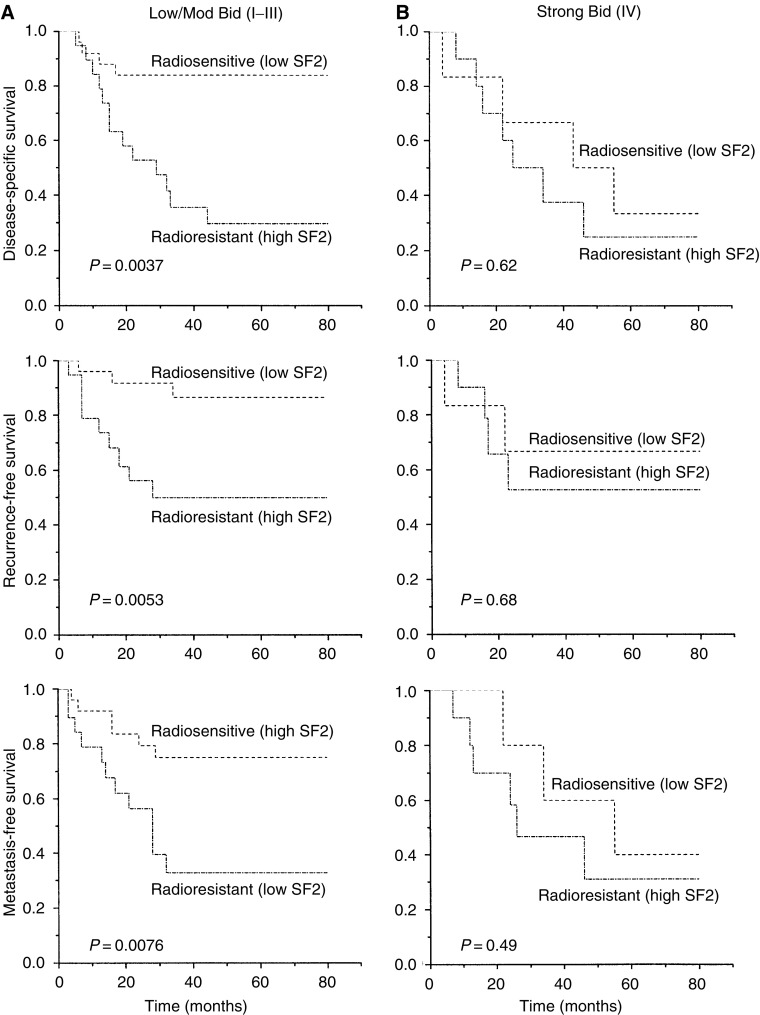
Bid expression and relationship with intrinsic tumour radiosensitivity (SF2) and treatment outcome. Patients (*n*=60) were stratified according to Bid expression and grouped according to intrinsic tumour radiosensitivity (SF2). (**A**) Low/Mod Bid (I–III); (**B**) Strong Bid (IV).

Bid expression was examined in relation to disease-specific, local recurrence-free and metastasis-free survival by Kaplan–Meier and log-rank analysis. A preliminary analysis revealed a trend for a worse survival for patients with strong Bid expression (category IV; Figure 3). Subsequent analyses were carried out, stratifying patients into two groups of low/moderate (categories I–III) and high (category IV) expression. High Bid expression was associated with a worse outcome following radiotherapy (Figure 3). In univariate analysis, disease stage and SF2 were the strongest prognostic factors for radiotherapy outcome (Table 2). Bivariate analysis was carried out to investigate the prognostic significance of Bid expression in patients stratified by median age (Figure 4), and it was found that the level of Bid expression in tumours was associated with a worse prognosis in younger patients. To further examine the relationship between Bid expression and poor prognosis following radiotherapy, patients were stratified according to Bid expression and survival was analysed according to tumour cell radiosensitivity, (SF2, Figure 5); in those patients with low/moderate tumour Bid expression, SF2 retained prognostic significance for disease-specific, recurrence-free and metastasis-free survival. However, for those patients whose tumours showed strong Bid expression, SF2 was not prognostic, with the outcome of treatment of the radiosensitive tumours becoming worse. In multivariate analysis, after allowing for stage, Bid expression was a significant prognostic factor for both disease-specific and metastasis-free survival, and had borderline significance for recurrence-free survival (Table 3).

## DISCUSSION

The relevance of the Bcl-2 apoptotic regulators as determinants for tumour progression and radiotherapy response in human tumours is unclear. Previous studies have been carried out on other Bcl-2 family members in different cancer types, but varied methodologies, investigational strategies and treatment protocols have confounded interpretation and comparison. This is the first study to investigate expression of Bid and its clinical significance for radiotherapy outcome in cervix carcinoma. Interpretation of the results is aided by the homogeneous radiation only treatment received by the patients in this study (before concurrent chemoradiotherapy was introduced) and the availability of complete long-term followup survival data.

Bid expression was widespread in normal and neoplastic epithelial cells of the cervix tissues, although heterogeneity was observed within and between individual tumour biopsies. The extent and intensity of Bid expression between tumour biopsies was compared and found to range widely, indicating substantially altered regulation of Bid protein levels within the carcinomas examined. Tumour Bid expression was higher in younger patients, and was related to a more aggressive tumour phenotype which was more likely to metastasise, but was not related to intrinsic tumour cell radiosensitivity. Notably, strong tumour Bid expression was prognostic for poor outcome, particularly for younger patients, and could provide additional outcome prediction information given tumour stage.

Variable expression of Bid was previously found in several types of human cancers other than cervix carcinoma, including neural tumours, colorectal adenocarcinomas, ovarian and prostate cancers (Krajewska *et al*, 2002). Regulation of Bid expression occurs via transcriptional and translational mechanisms and may directly reflect variable levels of apoptosis within the tumours. Conversely, altered regulation of Bid could occur via mutation, post-translational modification or aberrant sequestration. This could affect the protein half-life and activity of Bid, allowing an accumulation of Bid protein although its normal proapoptotic activity may be inhibited. It is therefore unclear whether strong Bid immunoreactivity and its association with an aggressive tumour type and poor prognosis is reflective of tumours with a high proliferative capacity and concurrent increased apoptotic levels, or due to defective function of Bid and reduced apoptotic potential. No correlations were found between the expression levels of Bid and previously determined measures of either apoptosis or proliferation to aid in this interpretation. For tumours within this cohort, a previous study has shown an association of increased apoptosis linked to poor prognosis (Levine *et al*, 1995). The 5-year survival rate and time to local recurrence was significantly reduced for patients with a tumour apoptotic index above the median. Apoptosis also showed a positive correlation with the mitotic index, indicating that apoptosis may be a reflection of tumour proliferation (Levine *et al*, 1995). Our finding that the tumour expression of the proapoptotic Bid is an adverse prognostic feature is consistent with this previous work. Other studies in cervical neoplasia have shown a positive correlation between proliferation and apoptosis (Isacson *et al*, 1996; Shoji *et al*, 1996; Tsang *et al*, 1999a, 1999b), and an association between high apoptosis and poor disease-free survival (Tsang *et al*, 1999a, 1999b). Although, in one study high apoptosis was associated with a good prognosis in early-stage disease (Wheeler *et al*, 1995), we have shown previously that the prognostic significance of apoptosis in cervix cancer is dependent on the tumour histology (Sheridan *et al*, 1999).

The increased prevalence for strong Bid expression in younger patients is indicative of a genetic influence underlying Bid overexpression, as patients who develop cancer earlier are more likely to be genetically predisposed. The prognostic significance determined specifically for younger women provides further indication that strong Bid expression is associated with the earlier development of more aggressive tumours that may be influenced genetically. Genetic factors which may lead to Bid overexpression may include direct mutations to the Bid gene, which result in enhanced half-life of Bid protein within cells. For example, mutation of Bid's ubiquitin acceptor sites results in a stabilised protein that is resistant to degradation (Breitschopf *et al*, 2000), and specific mutations within Bid BH-3 domain results in Bid protein that is defective in cytochrome release (Luo *et al*, 1998) and unable to interact with Bcl-2 or Bax (Wang *et al*, 1996). Mutations of Bid that affect the activity of the protein have also been reported in gastric tumours (Lee *et al*, 2004), and mutant Bid proteins, that mimic apoptotic defects observed in cells, have been exploited in *in vitro* studies (Wang *et al*, 1996; Kuwana *et al*, 2002; Letai *et al*, 2002). Tumourigenic aberrations to the post-translational control of Bid, such as its phosphorylation, cleavage or myristoylation, may affect its apoptotic potential (Desagher *et al*, 1999, 2001; Zha *et al*, 2000; Puthalakath and Strasser, 2002; Degli-Esposti *et al*, 2003).

A typical regulation or aberrant expression of other Bcl-2 family members may influence the level of Bid protein detectable within the cell and/or its functional ability. For example, Bcl-2 is commonly overexpressed in a variety of tumour types including cervix carcinoma (Chung *et al*, 2002), and has been shown to bind and sequester Bid to prevent its normal activity (Wang *et al*, 1996; Zong *et al*, 2001). Bax, Bak and Bcl-X_L_ (which also bind Bid) and caspases 3 and 8 (that directly affect Bid function) have also been reported to be differentially regulated in cervix carcinoma (Chung *et al*, 2002; Mukherjee *et al*, 2001). For this reason, assessment of the relative levels of proteins known to antagonise Bid could be valuable for the biopsies studied here. Alternatively, expression of Bid may be influenced by tumourigenic changes to cellular transcription factors such as p53, a known regulator of Bid mRNA transcription following overexpression and/or stabilisation of p53 after DNA damage (Sax *et al*, 2002). Interestingly, for a subset of the carcinomas studied here, the level of Bid expression was found to correlate weakly with p53 immunopositivity (*n*=42, *r*=0.32, *P*=0.041), indicating a link between expression of the two proteins. Recently, Bid was shown to be negatively regulated by the hypoxia-inducible transcription factor HIF-1 (Erler *et al*, 2004), and, as cervix carcinoma tumours are known to have variable hypoxic fractions (Nordsmark *et al*, 2001), it is possible that Bid expression may be linked to HIF-1 activity within tumours.

Tumour cells with other compensatory defects in apoptotic mechanisms may tolerate elevated levels of Bid and/or prevent the normal proapoptotic function of Bid. Elevated expression of apoptosis inhibitor proteins, such as XIAP, cIAP or survivin, might also allow downstream accumulation of Bid (Krajewska *et al*, 2003). Viral proteins expressed by certain human papilloma virus (HPV) types are aetiologically involved in the development of cervical cancer (Bosch *et al*, 1995; Zur Hausen, 2000) and may indirectly influence the expression or function of apoptotic regulators such as Bid. HPV type 16 has been shown to inhibit apoptotic signalling induced by death receptors (Aguilar-Lemarroy *et al*, 2001; Filippova *et al*, 2002), suggesting a method by which increased Bid protein levels may become dysregulated and tolerated by cells if upstream signalling becomes redundant.

If indeed Bid proapoptotic function is compromised, an attractive theory to explain increased metastatic potential may be the increased ability of tumour cells with reduced Bid function to avoid Fas/TNFR/Granzyme B-mediated apoptosis, induced by immune cells circulating in the body, thus enabling malignant cells to invade surrounding tissue early in tumour development. Similarly, defective Bid function could reduce the overall apoptotic potential in response to radiation and may partly explain increased rates of tumour recurrence following radiotherapy.

In conclusion, altered regulation of Bid expression within cervical carcinoma was shown and overexpression of Bid was an adverse prognostic factor for radiotherapy outcome. The relationship between strong Bid expression and poor outcome following radiotherapy was unrelated to *in vitro* measurement of intrinsic tumour cell radiosensitivity, but was associated with a more metastatic and aggressively malignant tumour phenotype in younger patients. Further study is required to examine the mechanism of Bid overexpression and its subsequent impact on apoptotic potential and disease phenotype. However, the results link Bid apoptotic signalling to tumour progression and treatment response, and may aid the development of tailored combined treatment and disease monitoring schedules for individual patients.

## Figures and Tables

**Table 1:** Summary of patient distribution according to Bid expression

		**Bid expression**				
**Parameter**	***n***	**1**	**2**	**3**	**4**	***P* value**
*Stage*						
I	32	12	6	7	7	
II	28	7	8	8	5	0.14
III	32	5	4	12	11	
IV	6	1	2	2	1	
						
*Differentiation*						
Well	18	8	3	5	2	
Moderate	58	12	11	17	18	0.095
Poor	17	4	6	3	4	
Unknown	5	1	0	4	0	
						
*Age (years)*						
⩽52	51	8	11	17	15	
>52	47	17	9	12	9	0.034
						
*SF2*						
Low (radiosensitive)	31	9	6	10	6	
High (radioresistant)	29	5	6	8	10	0.19

**Table 2:** Univariate log-rank analysis of putative prognostic factors for outcome following radiation therapy in cervix carcinoma

**Parameter**	***n***	**Disease-specific survival**	**Recurrence-free survival**	**Metastasis-free survival**
Stage	98	0.0001	0.068	0.0008
Differentiation	93	0.69	0.10	0.69
Age	98	0.87	0.70	0.24
SF2	60	0.0036	0.0093	0.0059
Bid expression	98	0.074	0.11	0.048

The *P*-values for each factor are given.

**Table 3:** Cox multivariate forward stepwise regression analysis to assess the independence and impact of variable factors on patient survival

**Parameter**	**Disease-specific survival**		**Recurrence-free survival**		**Metastasis-free survival**	
Stage	<0.0005	—	0.10	—	0.003	—
Age	0.87	0.54	0.76	0.96	0.25	0.36
Grade	0.69	0.60	0.12	0.053	0.69	0.81
SF2	0.005	0.003	0.015	0.018	0.009	0.013
Bid Expression	0.077	0.046	0.12	0.079	0.053	0.026

*P*-values for each factor are given. The left-hand columns give the *P*-value for each parameter derived from the multivariate analysis. The right-hand columns give *P*-values for each parameter after allowing for stage.
